# Diagnostic accuracy of the blood urea-to-creatinine ratio for differentiating AKI etiologies and dialysis prediction in adults: A systematic review and meta-analysis

**DOI:** 10.1016/j.clinsp.2026.101098

**Published:** 2026-09-09

**Authors:** Jia-Jin Chen, Tao-Han Lee, Yen-Ta Huang, Wen- Yu Ho, Ming-Jen Chan, Vin-Cent Wu, Ching-Chung Hsiao, Chih-Hsiang Chang

**Affiliations:** a Kidney Research Center, Department of Nephrology, Chang Gung Memorial Hospital, Linkou Branch, Taoyuan, Taiwan; b College of Medicine, Chang Gung University, Taoyuan, Taiwan; c Department of Nephrology, Chansn Hospital, Taoyuan, Taiwan; d Department of Surgery, National Cheng Kung University Hospital, College of Medicine, National Cheng Kung University, Tainan, Taiwan; e Department of Internal Medicine, National Taiwan University Hospital, Taipei, Taiwan, National Taiwan University Study Group on ARF (NSARF), Taiwan; f Department of Nephrology, New Taipei Municipal TuCheng Hospital, New Taipei City, Taiwan

**Keywords:** Acute kidney injury, Diagnostic accuracy, Pre-renal azotemia, Urea-creatinine ratio, Meta-analysis

## Abstract

**Background:**

The blood Urea-to-Creatinine Ratio (UCR) is one of the most readily available markers for differentiating the etiology of acute kidney injury and has long been emphasized in textbooks and review articles. Nevertheless, its diagnostic accuracy has not yet been systematically evaluated.

**Methods:**

We searched PubMed, Embase, and the Cochrane Library through May 2025. The diagnostic performance of the UCR for (1) Pre-renal azotemia/transient AKI and (2) Non-dialysis-requiring AKI was extracted and evaluated. A diagnostic accuracy meta-analysis was conducted using a bivariate model. We also compared the diagnostic accuracy of fractional excretion of sodium with UCR to predict pre-renal azotemia/transient AKI.

**Results:**

We identified 9 studies comprising 10 independent cohorts with a total of 16,412 participants. For pre-renal azotemia/transient AKI, the UCR demonstrated a pooled sensitivity of 0.74 (95% CI 0.43–0.91) and specificity of 0.52 (95% CI 0.21–0.82), with an AUC of 0.69. For non-dialysis-requiring AKI, the UCR showed a pooled sensitivity of 0.49 (95% CI 0.44–0.55) and specificity of 0.71 (95% CI 0.64–0.77), with an AUC of 0.63. Substantial heterogeneity was observed, and the certainty of evidence was rated low to very low.

**Conclusions:**

Based on currently available evidence, despite its long-standing role in textbooks and review articles, UCR demonstrates limited diagnostic accuracy for differentiating AKI etiologies and identifying non-dialysis-requiring AKI. Its clinical utility should be interpreted with caution given the substantial heterogeneity and low certainty of evidence, and it may be more appropriate to use UCR in combination with, or in place of, other traditional indices or novel biomarkers.

## Highlights

• First meta-analysis of UCR diagnostic accuracy in AKI.
• 9 studies (10 cohorts), 16,412 patients analyzed.
• High UCR for pre-renal AKI: AUC = 0.69, modest sensitivity, low specificity.
• High UCR for non-dialysis AKI: AUC = 0.63, limited discrimination.
• Findings support cautious use given heterogeneity and low-certainty evidence.

## Introduction

Acute Kidney Injury (AKI) is a frequent complication among critically ill patients and is associated with a substantial burden of both short- and long-term comorbidities as well as increased mortality.^1^, ^2^, ^3^ Acute kidney injury, traditionally, can be classified into pre-renal, intrinsic or post-renal etiologies.^4^ Among these categories, pre-renal azotemia is the most common, accounting for up to 70% of AKI in non-hospitalized settings.^5^ Clinical guidelines emphasize the importance of distinguishing pre-renal AKI from intrinsic renal AKI, as this differentiation can provide information for AKI management strategies.^6^ Early recognition of underlying etiologies, accurate risk stratification, timely fluid resuscitation, and adequate supportive care are all critical for improving renal outcomes.^4^, ^6^

Pre-renal AKI, also referred to as pre-renal azotemia, is characterized by a potentially reversible increase in serum creatinine due to reduced renal perfusion.^4^^,^^7^^,^^8^ In contrast to intrinsic causes such as acute tubular necrosis, pre-renal azotemia is typically expected to resolve within 24–48 h following appropriate volume repletion.^9^ Several diagnostic indices have been proposed to aid in the identification of pre-renal AKI. Textbooks and review articles commonly used blood urea nitrogen to creatinine ratio (BUN/Cr > 20), fractional excretion of sodium (FeNa < 1%), and elevated urine osmolality as key markers.^4^^,^^7^^,^^8^ Among these, the blood Urea-to-Creatinine Ratio (UCR) remains the most readily available and widely utilized tool. The concept of pre-renal azotemia associated with disproportionate elevations in blood urea relative to creatinine was first described by Fishberg et al.^10^^,^^11^ Carvounis et al. subsequently supported the diagnostic utility of an elevated UCR in a clinical cohort.^11^, ^20^ However, later studies have reported inconsistent results regarding its performance,^12^^,^^13^ and to date no systematic evaluation has comprehensively assessed the diagnostic accuracy of UCR for identifying pre-renal or transient AKI.

Therefore, we conducted a systematic review and meta-analysis to evaluate the diagnostic accuracy of an elevated UCR for: 1) Differentiating pre-renal azotemia or transient AKI from intrinsic or persistent AKI; 2) Identifying non-dialysis-requiring AKI; and 3) Comparing the diagnostic accuracy of UCR with that of FeNa.

## Material and methods

### Literature search strategy

The protocol of the current study was registered with PROSPERO (CRD420251117471). We conduct the current meta-analysis in accordance with Preferred Reporting Items for Systematic Reviews and Meta-Analyses (PRISMA) for Diagnostic Test Accuracy (DTA) Studies^14^ (Supplementary Protocol). Two independent reviewers (J.J. Chen and C.C. Hsiao) conducted a search for articles published before May 14, 2025 in PubMed, EMBASE and Cochrane Central. The literature search strategy was developed based on key concepts related to acute kidney injury, urea-to-creatinine ratio, and diagnostic accuracy. A combination of MeSH terms, free-text terms and related keywords (blood urea nitrogen to blood creatinine ratio, urea to creatinine ratio, acute kidney injury, acute renal failure, pre-renal acute kidney injury, intrinsic acute kidney injury, acute interstitial nephritis dialysis, etc.) were used. The detailed search strategy for each database is provided in Supplementary Table 1. Review articles and their relevant reference were also screened for potential studies. There were no limitations on language or article types. After removing duplicated articles by Rayyan, two reviewers (J.J. Chen and C.C. Hsiao) independently screened titles and abstracts. The full texts of the potentially relevant articles were then examined to decide whether the studies were eligible for inclusion.

### Study eligibility criteria

A study was eligible for inclusion if it involved adult patients with Acute Kidney Injury (AKI) or acute renal failure, defined either by established criteria or by pre-specified definitions. Additional eligibility criteria required that the study reported a cutoff value for the UCR and its diagnostic or predictive performance for at least one of the following outcomes: 1) Pre-renal azotemia/transient AKI versus intrinsic/persistent AKI, or 2) Non-dialysis-requiring AKI versus dialysis-requiring AKI. In cases of uncertainty or disagreement regarding study eligibility, a third reviewer (T.H. Lee) was consulted to reach consensus through discussion.

### Data extraction and outcome measurement

Two investigators (J.J. Chen and C.C. Hsiao) independently extracted relevant information from enrolled studies. We extracted data including first author, year of publication, study location, study design, patient source, AKI or acute renal failure definition, UCR cutoff value, definition of pre-renal azotemia/transient acute kidney injury vs. intrinsic acute kidney injury/persisted acute kidney injury, population mean age, female gender proportion, and inclusion and exclusion criteria. Regarding diagnostic performance, we extracted or calculated the number of true positives, false positives, true negatives and false negatives, and the number of outcomes of interest (Table 1, Table 2). When 2 × 2 contingency table data were not directly reported, they were reconstructed from available diagnostic performance measures, including sensitivity, specificity, and sample size, using standard methods or calculated directly from case series with available raw data.^15^ We considered patients with a higher UCR with pre-renal azotemia/transient AKI as true positives for the primary outcome. For the secondary outcome, a patient with a higher UCR with non-dialysis requiring AKI is considered a true positive. Disagreements about data extraction between the two authors (J.J. Chen and C.C. Hsiao) were resolved through discussion with a third author (Lee). We did not contact study authors for additional data.

**Table 1:** The characteristics of the included studies.

Study	Study Period	Design	Center	Country	Population	AKI criteria	Age (mean)	Gender (female%)
Dewitte, 2012	NR	Prospective	Single	France	ICU admission with AKI	RIFLE	66.6	21%
Fushimi, 1990	NR	Retrospective	Multicentre	Japan	Admission with acute azotemia	Pre-defined criteria (acute azotemia, acute elevation of serum creatinine to greater than 1.5 mg/dL)	61.4	50%
Manoeuvrier, 2017	2013–2014	Retrospective	Single	France	ER patients with elevated Cr > 1.5 mg/dL	KDIGO	75.7	37.20%
Rachoin, 2012 (derivation cohort)	NR	Retrospective	Single	U.S.	ICU admission with AKI	NR	64	40%
Rachoin, 2012 (validation cohort)	NR	Retrospective	Single	U.S.	ICU admission with AKI	NR	61	42%
Sakr, 2023	2021–2021	Case-controlled	Single	Egypt	ADHF admission with AKI	Pre-defined criteria (rise in absolute serum creatinine of 0.3 mg/dL or a 1.5 fold increase in serum creatinine levels within 48 h)	62.8	57%
Singer, 2012	NR	Prospective	Single	U.S.	Hospital admission with AKI	RIFLE	66.3	38%
Slikke, 2020	2016–2018	Prospective	Single	Netherlands	ER patients with infection + AKI	KDIGO	63	42%
Uchino, 2012	2000–2002	Retrospective	Single	Australia	Hospital admission with AKI	RIFLE	76.8	50.10%
Carvounis, 2002	NR	Prospective	Single	U.S.	Admission with acute renal failure	Pre-defined criteria (a rapidly increasing BUN > 30 mg/dL and creatinine > 1.5 mg/dL & serum creatinine increase in excess of 0.5 mg/dL in the preceding 2 days)	49	50%

AKI, Acute Kidney Injury; BUN, Blood Urea Nitrogen; ER, Emergent Room; ICU, Intensive Care Unit; KDIGO, Kidney Disease: Improving Global Outcomes; NR, Non-Reported; RIFLE, Risk, Injury, Failure, Loss of kidney function, and End-stage kidney disease classification.

**Table 2:** Diagnostic test performance of blood urea to creatinine for pre-renal azotemia/transient acute kidney injury or non-dialysis requiring Acute kidney injury.

Urea To Creatinine Ratio (UCR) for Pre-renal Azotemia/Transient Acute Kidney Injury Diagnosis													
Study	Cutoff value	Total Number		True Positive		True Negative	False Positive		False Negative		Timing Definition for Kidney Recovery	Outcome definition of pre-renal azotemia/AKI or transient AKI	
Carvounis, 2002	15	102		67		20	5		10		NR	Favor pre-renal: (1) History: volume depletion, decreased cardiac output, or vasodilation related to sepsis, liver failure, or anaphylaxis (2) No urine analysis result indicate ATN (3) Retrospective confirmation of response to treatment	
Dewitte, 2012	20	47		23		1	23		0		Within 3 days	Transient AKI: resolved within 3-days after inclusion with conventional treatment in the ICU	
Fushimi, 1990	20	14		4		6	0		4		Within 1‒3 days	Prerenal azotemia for renal failure (acute renal failure), based on the following criteria: (1) daily urine volume < 500 mL (2) development of renal failure in association with volume depletion (3) return of renal function to normal within 24‒72 h after correction of volume depletion (4) absence of cellular casts on urinalysis	
Manoeuvrier, 2017	20	1103		349		197	408		149		Within 7 days	Prerenal AKI: non-obstructive AKI with a return of plasma creatinine to 110% (or less) of baseline value during the 7 days following admission	
Singer, 2012	20	107		20		47	28		12		Within 3 days	Prerenal AKI: caused by factors that compromise renal perfusion, and when creatinine rapidly improved to baseline with volume repletion or improvement in cardiac output within 3 days of directed therapy	
Slikke, 2020	21	130		31		20	17		62		Before discharge	Transient AKI: Presence of AKI at the emergent department that was recovered at discharge (defined as a decrease in serum creatinine level to <50% or 0.3 mg/dL above baseline)	
**Urea To Creatinine Ratio (UCR) for Non-dialysis Requiring Diagnosis**													
**Study**	**Cutoff value**		**Total Number**		**True Positive**			**True Negative**		**False Positive**		**False Negative**	**Timing Definition for Dialysis**
Fushimi, 1990	20		14		4			5		0		5	NR
Rachoin, 2012 (derivation cohort)	25		1010		513			17		5		475	RRT within 30 days
Rachoin, 2012 (validation cohort)	25		10,228		4236			190		60		5742	RRT within 30 days
Sakr, 2023	17.4		30		14			1		2		13	NR
Uchino, 2012	20		3641		1885			97		52		1607	NR

AKI, Acute Kidney Injury; ATN, Acute Tubular Necrosis; NR, Non-Reported; RRT, Renal Replacement Therapy.

### Data synthesis and analysis

The total sample size and True Positive (TP), False Positive (FP), True Negative (TN), False Negative (FN) for UCR to predict pre-renal azotemia/transient AKI or non-dialysis AKI based on pre-defined cutoff value were extracted, or calculated from reported sensitivity, specificity. For the secondary outcome, a higher UCR was considered a positive test for identifying non-dialysis-requiring AKI; accordingly, the inverse interpretation applies for predicting dialysis requirement. We calculate the summary measures (pooled sensitivity, specificity, positive Likelihood Ratio [+LR], negative Likelihood Ratio [-LR] and Diagnostic Odds Ratio [DOR]) by a bivariate mixed-effects modeling framework through the *midas* package. The overall performance of UCR was assessed by the Area Under the Curve (AUC) of the summary Receiver Operating Characteristic (ROC) curve. Additionally, we used the likelihood ratio scattergram and Fagan plot to examine the clinical application of UCR as a diagnostic tool.

Heterogeneity examination was presented as the I^2^ index and p value of Chi-squared test. A *I*^2^ > 50% was considered to indicate substantial heterogeneity. The publication bias was assessed by the Deek funnel plot and test.^16^ Subgroup analysis was performed to examine whether there is a difference in the diagnostic performance in different subgroups (BCR cutoff value 20 vs. other; study design prospective vs. retrospective; exclusion of CKD or not). Three studies also reported the diagnostic accuracy of FeNa for pre-renal azotemia/transient AKI. Accordingly, we performed a meta-regression including test type (UCR vs. FeNa) as a covariate to directly compare their diagnostic performance. Subgroup analyses were conducted using univariable meta-regression with the *midas* package, and direct comparisons between FeNa and UCR were performed using the *metadta* package. A sensitivity analysis was conducted excluding studies with a case-control design or those using a Youden index-derived cutoff value. All other mentioned analyses were performed via *midas* package. All analyses were performed using Stata version 19 (2025).^17^

### Risk of bias assessment

The risk of bias was assessed by the Quality Assessment of Diagnostic Accuracy Studies 2 (QUADAS-2) tool.^18^ A domain is rated as low risk of bias only when all signaling questions receive “yes” responses. If even one question is answered “no,” the domain is classified as high risk of bias. The judgment of “applicability” followed the same principle as in the bias section. Two independent reviewers (J.J. Chen and C.C. Hsiao) rated high, low or unclear risk for four domains and disagreements between the reviewers were resolved by discussion with another author (Huang). The overall quality of evidence for the diagnostic performance of the UCR for pre-renal azotemia/transient AKI or non-dialysis requiring AKI was evaluated based on the guidelines of the GRADE Working Group methodology.^19^

## Results

### Search results and study characteristics

PRISMA flowchart is provided (Supplementary Fig. 1). The electronic database search identified a total 796 potentially eligible studies from PubMed, 1758 from EMBASE, 170 from Cochrane Library. After removing duplicate articles, the remaining 1943 articles were screened. After screening the titles and abstracts, the full texts of 21 studies were reviewed to assess their eligibility. An additional five studies were identified from the references of related articles. After excluding studies for various reasons (no outcome of interest [n = 12]; not AKI population [n = 6] (Supplementary Table 2), 9 studies (10 separate cohorts) comprising 16412 patients were included for analysis.^13^^,^^15^^,^^20^, ^21^, ^22^, ^23^, ^24^, ^25^, ^26^ The enrolled patients had a mean age ranging from 49- to 76.8-years and 43.3% were female participants. 4 of 9 studies were prospective cohorts, 4 were retrospective and the remaining 1 was case-control in design. 3 of the enrolled studies defined AKI by RIFLE criteria, 3 pre-defined criteria and 2 KDIGO criteria and one study (Rachoin, 2012) did not report the AKI criteria.^22^ Patients' sources varied from emergency department patients, hospitalized patients and ICU admission patients.

### Pre-renal azotemia or transient acute kidney injury

**Fig. 1:**
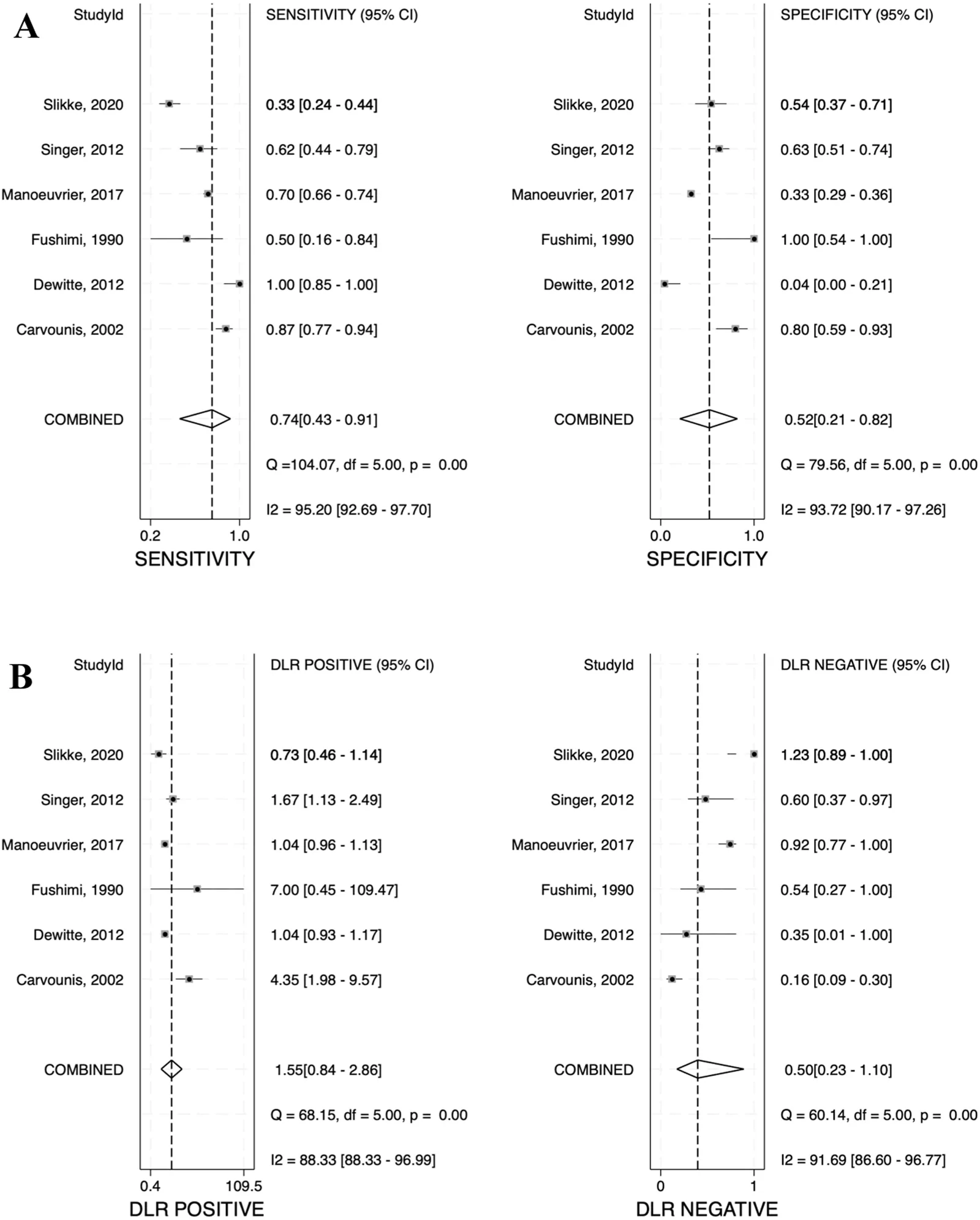
Forest plot of blood UCR diagnostic accuracy for pre-renal azotemia/transient AKI: (A) Sensitivity & specificity, (B) Positive likelihood ratio and negative likelihood ratio. (A) Forest plots of study-level sensitivity and specificity with 95% CIs. Diamonds denote the bivariate random-effects pooled estimates. Dashed vertical lines indicate pooled point estimates. (B) Forest plots of positive and negative diagnostic likelihood ratios with 95% CIs. Symbols as in panel A. AKI, Acute Kidney Injury; DLR, Diagnostic Likelihood Ratio; UCR, urea to creatinine ratio.

**Fig. 2:**
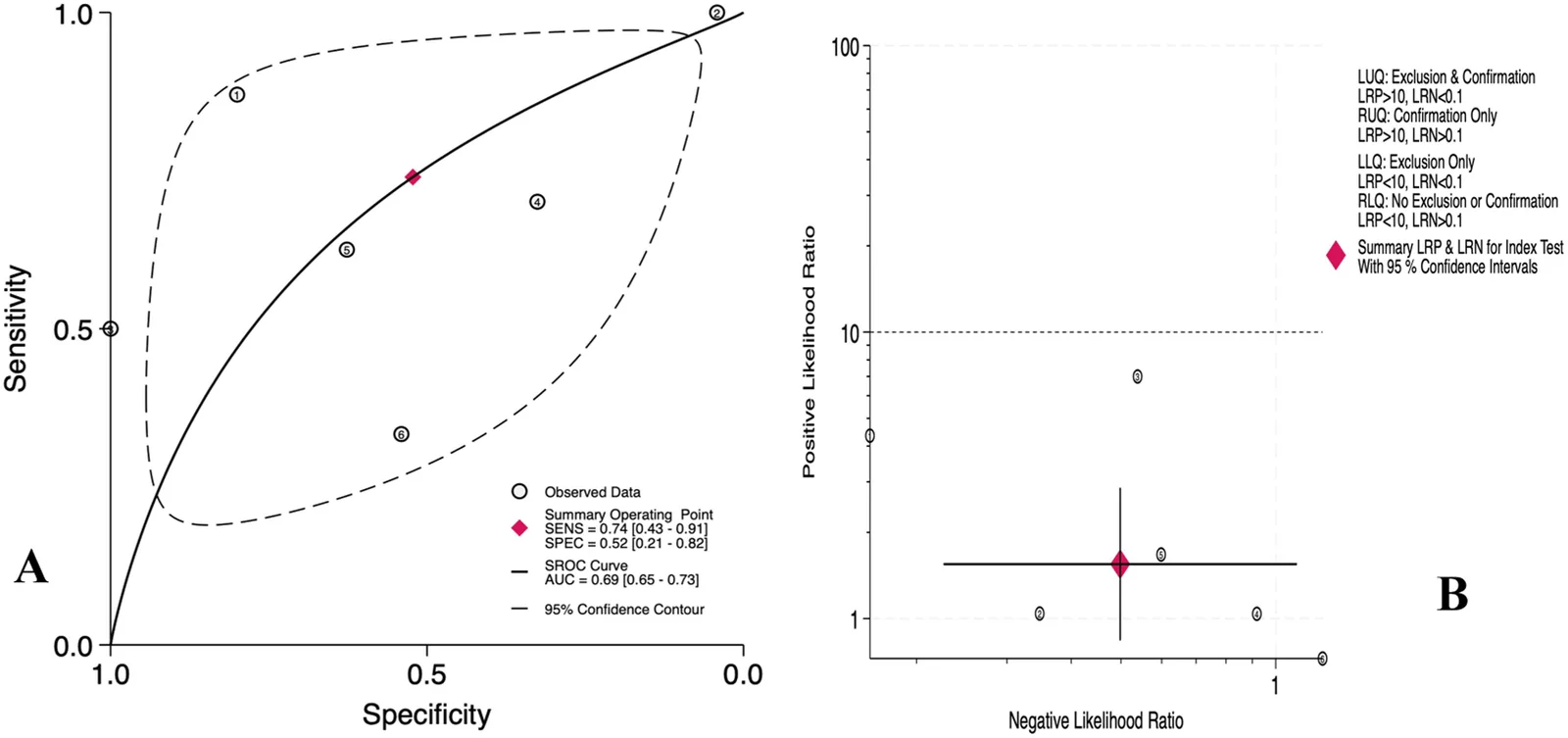
SROC curves (A) and likelihood ratio scattergram (B) of blood UCR for prediction of pre-renal azotemia/transient AKI. (A) Summary receiver-operating characteristic (SROC) curve with 95% confidence contour. The red diamond marks the summary operating point. Open circles are study estimates. (B) Likelihood-ratio scattergram. Dashed reference lines indicate ideal “rule-in” (upper-right) and “rule-out” (lower-left) thresholds; the pooled point falls in the no-confirmation/no-exclusion zone. AKI, Acute Kidney Injury; UCR, urea to creatinine ratio; SROC, summary receiver operating characteristic.

The diagnostic value, cutoff points from enrolled studies are summarized in Table 2. The pooled sensitivity and specificity were 0.74 (95% CI 0.43–0.91) and 0.52 (95% CI 0.21–0.82), respectively with substantial heterogeneity (*I*^2^ = 95.2, 95% CI 92.69‒97.7 for sensitivity and *I*^2^ = 93.72, 95% CI 90.17‒97.26 for specificity) (Fig. 1A). The pooled positive LR and negative LR were 1.55 (95% CI 0.84–2.86) and 0.50 (95% CI 0.23–1.10), respectively with substantial heterogeneity (Fig. 1B). The Area Under the Curve (AUC) for SROC to summarize diagnostic accuracy was 0.69 (95% CI 0.65–0.73) (Fig. 2A). The likelihood ratio scattergram showed the pooled positive, negative LR within the right lower quarter indicated UCR is not an ideal diagnostic tool for exclusion or inclusion (Fig. 2B). One study (Carvounis, 2002) used pre-renal azotemia criteria without clearly stating about transient AKI/renal recovery.^20^ After excluding this study, the pooled sensitivity and specificity were 0.67 (95% CI 0.32–0.90) and 0.41 (95% CI 0.14–0.74), respectively with substantial heterogeneity (Supplementary Fig. 2). The pooled positive LR and negative LR were 1.15 (95% CI 0.85–1.55) and 0.79 (95% CI 0.47–1.33), respectively with substantial heterogeneity (Supplementary Fig. 3). The Area Under the Curve (AUC) for SROC to summarize diagnostic accuracy was 0.56 (95% CI 0.52–0.60) (Supplementary Fig. 4) and the likelihood ratio scattergram showed UCR is not a good diagnostic tool for predicting renal recovery (Supplementary Fig. 5).

### Non-dialysis requiring acute kidney injury

**Fig. 3:**
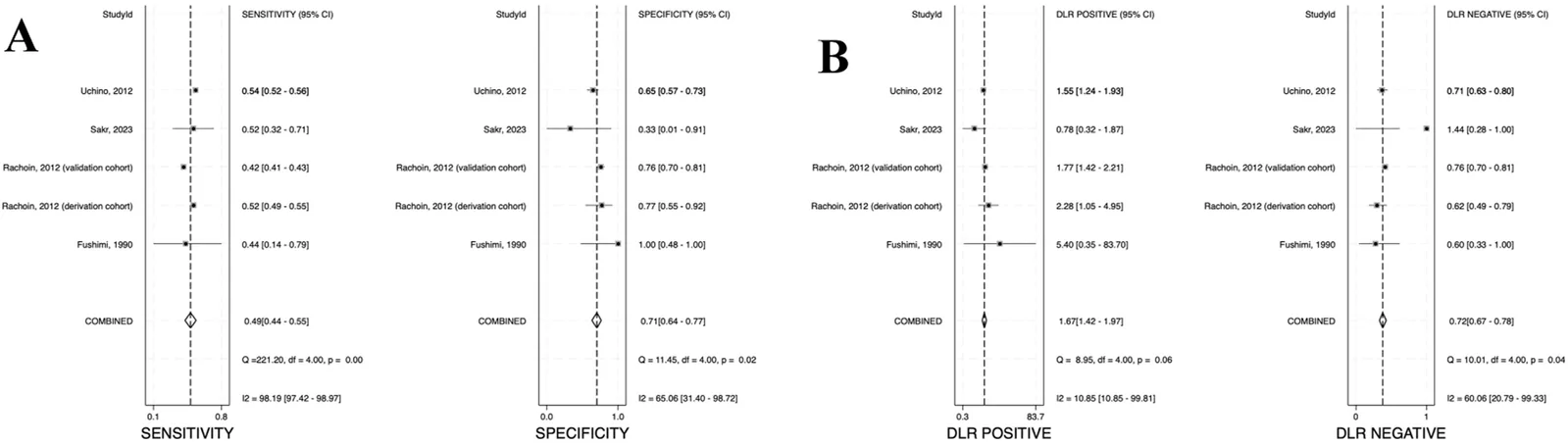
Forest plot of blood UCR diagnostic accuracy for non-dialysis requiring AKI: (A) Sensitivity & specificity, (B) Positive likelihood ratio and negative likelihood ratio. (A) Forest plots of study-level sensitivity and specificity with 95% CIs. Diamonds denote the bivariate random-effects pooled estimates. Dashed vertical lines indicate pooled point estimates. (B) Forest plots of positive and negative diagnostic likelihood ratios with 95% CIs. Symbols as in panel A. AKI, acute kidney injury; DLR, diagnostic likelihood ratio; UCR, Urea to creatinine ratio.

**Fig. 4:**
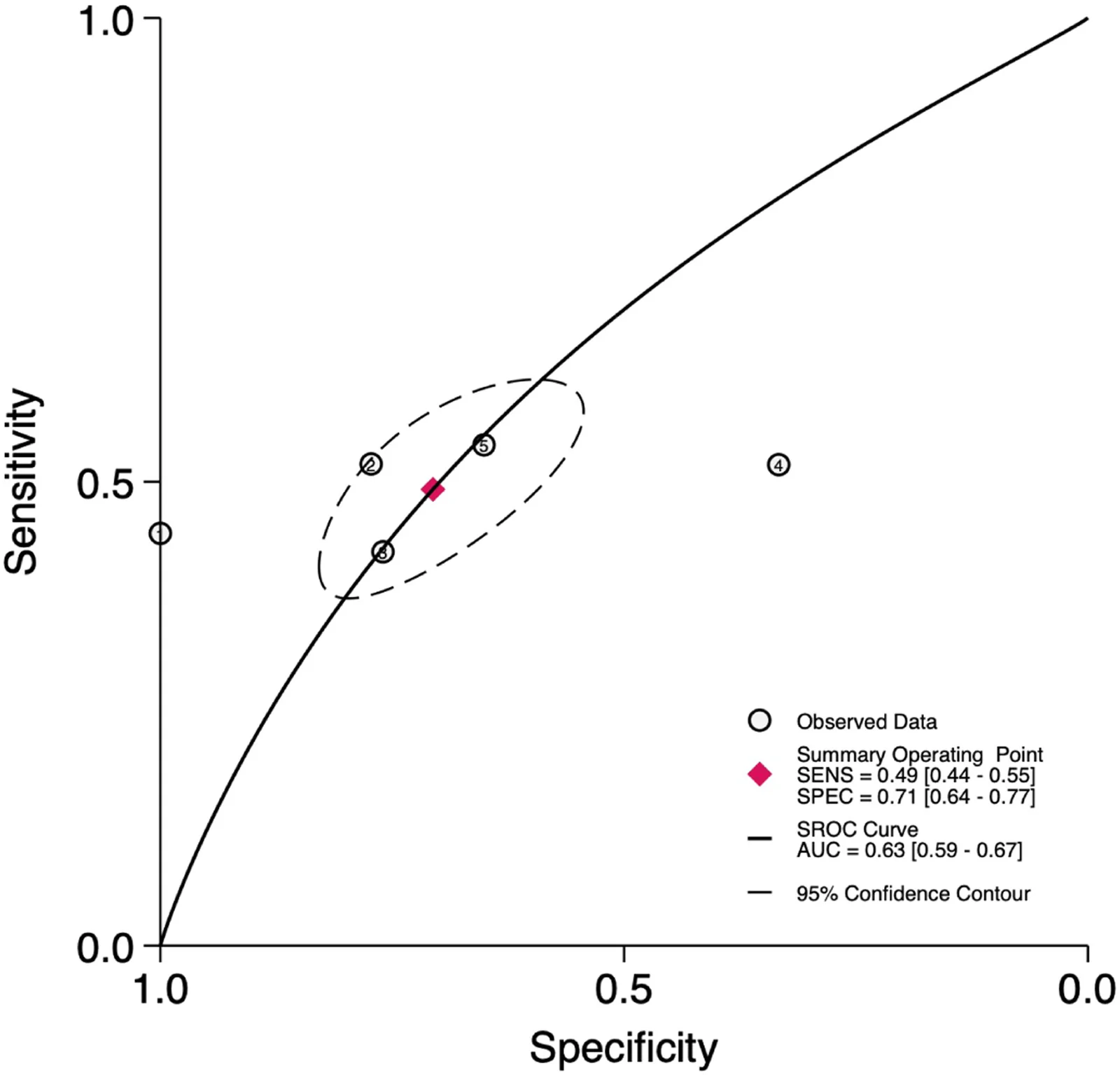
SROC curves of blood UCR for prediction of non-dialysis requiring AKI. Summary Receiver-Operating Characteristic (SROC) curve with 95% confidence contour. The red diamond marks the summary operating point. Open circles are study estimates. AKI, Acute Kidney Injury; UCR, Urea to creatinine ratio; SROC, summary receiver operating characteristic.

The pooled sensitivity and specificity were 0.49 (95% CI 0.44–0.55) and 0.71 (95% CI 0.64–0.77), respectively with substantial heterogeneity (Fig. 3A). The pooled positive LR and negative LR were 1.67 (95% CI 1.42–1.97) and 0.72 (95% CI 0.67–0.78), respectively with substantial heterogeneity (Fig. 3B). These findings suggest that a higher UCR is associated with a lower likelihood of requiring dialysis, although the overall discriminatory ability remains limited. The Area Under the Curve (AUC) for SROC to summarize diagnostic accuracy was 0.63 (95% CI 0.59–0.67) (Fig. 4) and the likelihood ratio scattergram showed UCR is not an google diagnostic tool for predicting non-dialysis requiring AKI from dialysis-requiring AKI (Supplementary Fig. 6).

### Subgroup analysis & sensitivity analysis

The Spearman correlation coefficient test yielded a p-value of 0.66, indicating no statistically significant evidence of a threshold effect. We further conducted subgroup analyses based on UCR cutoff (20 vs. other) and study design (prospective vs. retrospective). Sensitivity and specificity were similar across different UCR cutoff values (p = 0.39 and 0.42, respectively). Likewise, no significant differences in diagnostic indices were observed between prospective and retrospective studies, between studies excluding CKD population or not (Supplementary Table 3). Sensitivity analysis was performed after excluding the study with a case-control design.^23^ For predicting non-dialysis-requiring AKI, a higher UCR showed a pooled sensitivity of 0.49 (95% CI 0.43–0.54) and specificity of 0.71 (95% CI 0.64–0.77). The pooled positive and negative likelihood ratios were 1.70 (95% CI 1.40–2.00) and 0.72 (95% CI 0.67–0.78), respectively, which were consistent with the primary analysis (Supplementary Fig. 7 and 8).

### Comparison of FeNa with UCR

Only three studies provided a direct comparison of the diagnostic accuracy of FeNa and UCR. The available data do not allow a definitive conclusion regarding their comparative performance. FeNa showed a relative sensitivity of 0.77 (95% CI 0.45–1.33) and a relative specificity of 1.33 (95% CI 0.50–3.57) (Supplementary Fig. 9 and 10). Given the small number of studies and wide confidence intervals, the current evidence is insufficient to determine whether FeNa and UCR differ in diagnostic accuracy.

### Risk of bias (RoB) of enrolled studies and certainty of evidence (CoE)

The publication bias assessment using Deeks’ funnel plot did not identify clear evidence of publication bias (p = 0.19 and 0.7 for pre-renal azotemia/transient AKI and non-dialysis requiring AKI, respectively; Supplementary Figs. 11 and 12). However, the ability to detect publication bias was limited by the small number of included studies.

Using the QUADAS-2 tool, potential biases were identified. In Domain 1, assessing patient selection, the study by Sakr et al. was considered as high risk due to case-control design.^23^ In Domain 2, the study by Sakr et al. also considered as high risk due to applying the cutoffs defined by the Youden index.^23^ Other detailed information for risk of bias assessment is provided (Supplementary Table 4 and Supplementary Fig. 13).

The Certainty of Evidence (CoE) for the diagnostic performance of UCR in identifying pre-renal azotemia/transient AKI was rated as low, while the CoE for non-dialysis-requiring AKI was rated as very low. Downgrading was primarily due to substantial heterogeneity without an identifiable source and the inclusion of one study (Sakr et al.)[23] judged to be at high risk of bias. The detailed results of the CoE assessment are summarized in Supplementary Tables 5 and 6.

### Fagan plot analysis

To demonstrate the clinical implications of UCR as a diagnostic tool, we applied Fagan’s nomogram using different pre-test probabilities. For pre-renal azotemia/transient AKI (pre-test probability 45% and 55%), the post-test probabilities showed only modest changes after a positive or negative test, indicating limited clinical utility Supplementary Figure 14. For non-dialysis-requiring AKI (pre-test probability 85% and 95%), the shifts in post-test probability were also small, further suggesting that UCR offers minimal diagnostic value Supplementary Figure 15.

## Discussion

Our study highlighted several key findings: 1) An elevated UCR demonstrated a pooled sensitivity of 0.74 and a pooled specificity of 0.52 for pre-renal azotemia/transient AKI, with an overall diagnostic accuracy reflected by an AUC of 0.69; 2) An elevated UCR was not accurate in distinguishing non-dialysis-requiring AKI from severe, dialysis-requiring AKI, showing a pooled sensitivity of 0.49, a pooled specificity of 0.71, and an overall AUC of 0.63.

### Physiological basis and limitations of the BUN/Creatinine ratio

The BUN/Cr ratio has long been used as an adjunctive diagnostic tool in AKI patients.^27^^,^^28^ Several conditions could result in impaired normal kidney compensatory mechanisms for adjusting kidney perfusion, leading to decreased renal perfusion. For example, intrinsic factors that result in afferent arteriolar vasoconstriction like hepatorenal syndrome, sepsis and extrinsic factors like cyclosporine/tacrolimus exposure.^29^ In pre-renal AKI status, the renal perfusion is reduced, which enhances tubular urea reabsorption, leading to a disproportionately elevated BUN relative to creatinine, typically yielding a ratio > 20:1. In intrinsic renal injury conditions, such as acute tubular necrosis, characterized by impaired tubular reabsorptive capacity, resulting in a normal or reduced ratio.^13^^,^^29^ However, lots of extrarenal factors can influence the BUN/Cr ratio, such as protein intake, catabolic states (for example, glucocorticoid exposure or hyperthyroidism) and gastrointestinal bleeding.^29^

### Comparison with established and emerging diagnostic tools

Several diagnostic tools for differentiating AKI etiologies have been reported. For example, the fractional excretion of sodium (FeNa < 1%), a long-standing simple marker, showed pooled sensitivity and specificity of 90% and 82% for distinguishing pre-renal from intrinsic AKI.^30^ However, its accuracy declined in patients with chronic kidney disease or on diuretics (sensitivity 83%, specificity 66%).^30^ Novel biomarkers demonstrate better performance: urinary calprotectin showed pooled sensitivity and specificity of 0.90 and 0.93,^31^ while urine NGAL at 650 ng/mL differentiated intrinsic/ATN from hepatorenal syndrome in cirrhosis with sensitivity 1.0, specificity 0.84, and AUC = 0.95;^32^ a meta-analysis reported pooled sensitivity 0.86 and specificity 0.82 for the same setting.^33^ Sonographic assessment of renal resistive index (RI > 0.8) also demonstrated high sensitivity (92%) and specificity (85%) for persistent AKI/ATN.^34^^,^^35^ Taken together, compared with these established and novel tools, our findings indicate that UCR is not a reliable diagnostic marker for pre-renal azotemia/transient AKI and should be interpreted with caution.

### Impact of heterogeneity and clinical interpretation

Importantly, the substantial heterogeneity observed across studies has important implications for the interpretation of our pooled estimates. First, the UCR cutoff values varied considerably (ranging from approximately 15 to 25). Although the Spearman test did not indicate a statistically significant threshold effect, the potential influence of varying cutoff values on the trade-off between sensitivity and specificity should still be considered. Second, the definitions of pre-renal azotemia and transient AKI, the diagnostic criteria and recovery time windows were not uniform, which might potentially lead to outcome misclassification. Third, the included populations were derived from heterogeneous clinical settings. Taken together, these sources of heterogeneity suggest that the pooled diagnostic estimates should be interpreted cautiously, as they may not represent a single, consistent underlying test performance. These problems contributed to the observed low certainty of evidence and limit the generalizability of our findings to specific clinical contexts.

Another source of heterogeneity relates to the reference standard used to define pre-renal azotemia or transient AKI. Notably, there is no universally accepted gold standard for defining pre-renal or transient AKI. In the included studies, these conditions were commonly defined based on clinical etiologic assessment in conjunction with short-term recovery of serum creatinine following treatment, with varying time windows (e.g., 24–72 h, up to 7-days, or at hospital discharge). Prior work by Kellum et al. showed that early reversal of AKI is associated with improved outcomes^36^ compared with late reversal; however, early recovery does not necessarily reflect a true pre-renal etiology, as improvement may result from treatment effects, hemodynamic stabilization, or resolution of concurrent insults. Differences in assessment timing and clinical context may further lead to misclassification, potentially biasing the estimated diagnostic accuracy.

From a clinical perspective, the use of non-dialysis-requiring AKI as the target condition may be less intuitive than directly predicting dialysis requirement. In this context, our findings can be interpreted inversely, whereby a higher UCR is associated with a lower probability of requiring renal replacement therapy. However, given the modest diagnostic performance (AUC = 0.63) and substantial heterogeneity, UCR does not appear to be a reliable tool for predicting dialysis requirement in clinical practice.

### Potential prognostic role of UCR

Additionally, we highlight the potential role of UCR as a prognostic marker in various clinical settings. Slikke et al. reported that AKI patients with elevated UCR had a higher risk of mortality, with an adjusted Hazard Ratio (HR) of 2.0.^25^ A recently published meta-analysis by Paulus et al.^37^ also demonstrated that patients with UCR ≥20, compared with those with UCR < 20, had an increased risk of in-hospital mortality (adjusted HR = 1.29). Moreover, UCR has also demonstrated to be associated with persistent critical illness and ICU admission.^37^

### Limitation & strength

Although the UCR has long been emphasized in textbooks and reviews, and prior studies have yielded conflicting results, no meta-analysis had systematically evaluated its diagnostic accuracy for differentiating AKI etiologies. Our study is also the first to assess its ability to distinguish non-dialysis-requiring AKI from severe, dialysis-requiring AKI. Several limitations warrant consideration. First, the included studies exhibited substantial heterogeneity, and some have been published more than two decades ago, during which diagnostic practices and definitions likely evolved. The limited number of eligible studies further constrained detailed subgroup analyses to explore sources of heterogeneity. Second, key clinical subgroups with atypically low creatinine (e.g., cirrhosis or cachexia) or high urea (e.g., gastrointestinal bleeding or glucocorticoid exposure) were not reported, leaving the diagnostic accuracy of UCR in these populations uncertain. Third, definitions of pre-renal/transient AKI and recovery windows, as well as UCR cutoffs, varied across studies, introducing potential threshold effects. Finally, head-to-head comparisons with FeNa were available in only three studies, limiting the precision of between-test comparisons.

## Conclusions

In summary, the blood Urea-to-Creatinine Ratio (UCR) demonstrates limited diagnostic accuracy for distinguishing pre-renal azotemia or transient AKI and for identifying non-dialysis-requiring AKI in adults. Heterogeneity and lack of standard definition of pre-renal azotemia or transient AKI was noted. Therefore, despite its long-standing use as an accessible clinical tool, its utility should be interpreted with caution. UCR may be better used in conjunction with other clinical indices or emerging biomarkers rather than as a standalone diagnostic test. Larger, well-designed studies incorporating standardized definitions and physiologic assessments are needed to better define its role in AKI management.

## Ethics approval and consent to participate

Not applicable.

## Consent for publication

Not applicable.

## Data availability

The datasets used and/or analyzed during the current study are available from the corresponding author on reasonable request.

## Declaration of generative AI

We used ChatGPT for English editing.

## Authors’ contributions

All authors participated in the research and preparation of the manuscript. Conceptualization: J.J. Chen, V.C. Wu, Y.T. Huang, C.H. Chang; Data curation: J.J. Chen, T.H. Lee, C.C. Hsiao; Formal analysis: J.J. Chen, C.H. Chang, C.C. Hsiao, Y.T. Huang; Funding acquisition: C.H. Chang, V.C. Wu, Y.T. Huang; Investigation: J.J. Chen, T.H. Lee, C.C. Hsiao; Methodology: J.J. Chen, C.H. Chang, C.C. Hsiao, Y.T. Huang; Project administration: C.H. Chang, V.C. Wu, Y.T. Huang; Writing-original draft: J.J. Chen, T.H. Lee, C.H. Chang; Writing-review and editing: W.Y. Ho, M.J. Chan, L.Y, Ma, V.C. Wu; Visualization & Table Creation: W.Y. Ho, M.J. Chan, L.Y, Ma; Validation: W.Y. Ho, M.J. Chan, L.Y, Ma. All authors read and approved the final manuscript.

## Funding

This research received no external funding. This work was supported by grants from Chang Gung Memorial Hospital (CMRPG5M0111) and from Taiwan National Science and Technology Council (NSTC 155-2314-B-182A-111).

## Conflicts of interest

The authors declare no conflicts of interest.
