# Supplementary material for: Diagnostic accuracy of the blood urea-to-creatinine ratio for differentiating AKI etiologies and dialysis prediction in adults: A systematic review and meta-analysis
Source: Clinics (Sao Paulo). 2026 Sep 9;81:101098. doi: 10.1016/j.clinsp.2026.101098 (PMC13573689; doi:10.1016/j.clinsp.2026.101098)
Supplement: Supplementary file 1 [file mmc1.docx]

**CLINICS-D-25-02068**

**Supplementary Profile**

**Supplementary Protocol:** PRISMA checklist

**Supplementary Table Legend**

**Supplementary Table 1.** Search Strategy and Result

**Supplementary Table 2.** Reasons for Excluding Full-text Screening Studies

**Supplementary Table 3.** Subgroup analysis

**Supplementary Table 4.** QUADAS assessment

**Supplementary Table 5.** Summary of Certainty of Evidence Assessment for pre-renal azotemia/transient AKI

**Supplementary Table 6.** Summary of Certainty of Evidence Assessment for non-dialysis requiring AKI

**Supplementary Figure Legend**

**Supplementary Figure 1.** PRISMA flow chart

**Supplementary Figure 2.** Forest plot of UCR diagnostic accuracy for transient AKI: sensitivity and specificity

**Supplementary Figure 3.** Forest plot of UCR diagnostic accuracy for transient AKI: positive likelihood ratio and negative likelihood ratio

**Supplementary Figure 4.** SROC curves of Blood UCR for prediction of Transient AKI

**Supplementary Figure 5.** Likelihood ratio scattergram of UCR for transient AKI

**Supplementary Figure 6.** Likelihood ratio scattergram of UCR for non-dialysis requiring AKI

**Supplementary Figure 7.** Sensitivity and specificity of UCR for non-dialysis requiring AKI: Sensitivity Analysis

**Supplementary Figure 8.** Positive likelihood ratio and negative likelihood ratio of UCR for non-dialysis requiring AKI: Sensitivity Analysis

**Supplementary Figure 9.** Forest plot of UCR vs FeNA for pre-renal azotemia/transient AKI

**Supplementary Figure 10.** SROC curves of Blood UCR vs FeNA for prediction of Pre-renal Azotemia/Transient AKI

**Supplementary Figure 11.** Funnel plot for pre-renal azotemia/transient AKI

**Supplementary Figure 12.** Funnel plot for non-dialysis requiring AKI

**Supplementary Figure 13.** Summary of risk of bias assessment

**Supplementary** **Figure 14.** Fagan's Nomogram for Blood UCR as Pre-renal Azotemia/Transient AKI Prediction Marker with Pre-test probability 45% (A) and 55% (B)

**Supplementary** **Figure 15.** Fagan's Nomogram for Blood UCR as Non-dialysis Requiring AKI Prediction Marker with Pre-test probability 85% (A) and 95% (B)

**Supplementary Table 1. Search Strategy and Result**

**Pubmed through May 14^st^, 2025**

#1	(((((Blood urea nitrogen to blood creatinine ratio) OR (Urea to creatinine ratio)) OR (BUN to Cr ratio)) OR (Urea-Creatinine Ratio)) OR (urea-to-creatinine ratio)) OR (BCR)

#2	(((((((Pre-renal acute kidney injury) OR (intrinsic acute kidney injury)) OR (acute interstitial nephritis)) OR (acute tubulointerstitial nephritis)) OR (acute tubular necrosis)) OR (acute kidney injury)) OR (acute renal failure)) OR (dialysis)

#3	((((((Blood urea nitrogen to blood creatinine ratio) OR (Urea to creatinine ratio)) OR (BUN to Cr ratio)) OR (Urea-Creatinine Ratio)) OR (urea-to-creatinine ratio)) OR (BCR)) AND ((((((((Pre-renal acute kidney injury) OR (intrinsic acute kidney injury)) OR (acute interstitial nephritis)) OR (acute tubulointerstitial nephritis)) OR (acute tubular necrosis)) OR (acute kidney injury)) OR (acute renal failure)) OR (dialysis)) Filters: Humans, Adult: 19+ years

**Result: 796**

**EMbase through May 14^st^, 2025**

#1	urea AND to AND creatinine AND ratio

#2	bun AND to AND cr AND ratio

#3	urea creatinine AND ratio

#4	urea AND nitrogen AND to AND blood AND creatinine AND ratio

#5	urea to creatinine' AND ratio

#6	urea creatinine ratio

#7	'pre renal' AND acute AND kidney AND injury

#8	'acute kidney failure'

#9	acute AND kidney AND injury

#10 'interstitial nephritis'

#11 acute AND interstitial AND nephritis

#12 'acute tubulointerstitial nephritis'

#13 'acute kidney tubule necrosis'

#14 'dialysis'

#15 'renal replacement therapy'

#16 #1 OR #2 OR #3 OR #4 OR #5 OR #6

#17 #7 OR #8 OR #9 OR #10 OR #11 OR #12 OR #13 OR #14

#18 #16 AND #17

#19 #18 AND 'human'/de AND [adult]/lim

**Result: 1758**

**Cochrane library through May 14^st^, 2025**

#1	(Blood urea nitrogen to blood creatinine ratio):ti,ab,kw OR (Urea to creatinine ratio):ti,ab,kw OR (BUN to Cr ratio):ti,ab,kw OR (Urea-Creatinine Ratio):ti,ab,kw OR (urea-to-creatinine ratio):ti,ab,kw (Word variations have been searched)

#2	MeSH descriptor: [Acute Kidney Injury] this term only

#3	acute renal failure

#4	acute kidney injury

#5	MeSH descriptor: [Renal Dialysis] explode all trees

#6	(Pre-renal acute kidney injury):ti,ab,kw OR (intrinsic acute kidney injury):ti,ab,kw OR (acute interstitial nephritis):ti,ab,kw OR (acute tubulointerstitial nephritis):ti,ab,kw OR (acute tubular necrosis):ti,ab,kw (Word variations have been searched)

#7	#2 OR #3 OR #4 OR #5 OR #6

#8	#1 AND #7

**Result 170 articles**

**Supplementary Table 2. Reasons for Excluding Full-text Screening Studies**

| Number | First author, Year | Title | Reason for exclusion |
| --- | --- | --- | --- |
| 1 | Bell, 1947 | EXTRARENAL AZOTEMIA AND TUBULAR DISEASE |  |
| 2 | Buckenmayer, 2023 | Evaluation of simple diagnostic parameters in acute kidney injury in hospitalized patients—diagnostic recommendations for non‑nephrologists |  |
| 3 | Casas-Aparicio, 2024 | Persistent Increase in the Blood Urea Nitrogen/Creatinine Index (PI- BUN/Cr) Phenotypes the Clinical Course of Patients with Severe COVID- 19 |  |
| 4 | Feinfeld, 2002 | Massive and disproportionate elevation of blood urea nitrogen in acute azotemia |  |
| 5 | Hanson, 2011 | Laboratory prediction of the requirement for renal replacement in acute falciparum malaria |  |
| 6 | İnal, 2014 | Utility of serum creatinine/cystatin C ratio in diagnosis of postrenal acute kidney injury |  |
| 7 | Kahn, 1972 | The Significance of Serum Creatinine and the Blood Urea/Serum Creatinine Ratio in Azotaemia |  |
| 8 | Lievre, 2023 | Urine‑to‑plasma creatinine ratio and fractional excretion of urea performance in acute‑on‑chronic kidney disease patients |  |
| 9 | Moledina, 2022 | Development and external validation of a diagnostic model for biopsy proven acute interstitial nephritis using electronic health record data |  |
| 10 | Morgan, 1997 | Plasma creatinine and urea: creatinine ratio in patients with raised plasma urea | No Outcome of Interest |
| 11 | Pons, 2013 | Diagnostic accuracy of early urinary index changes in differentiating transient from persistent acute kidney injury in critically ill patients: multicenter cohort study |  |
| 12 | Takaya, 2015 | Risk Stratification of Acute Kidney Injury Using the Blood Urea Nitrogen/Creatinine Ratio in Patients With Acute Decompensated Heart Failure | No Outcome of Interest |
| 13 | Li, 2025 | Blood Urea Nitrogen/Creatinine Ratio (BUR): A Novel Predictor of Early Prognosis in Acute Type A Aortic Dissection | Not AKI population |
| 14 | Nangalia, 2019 | Urea to creatinine ratio and its change is a powerful and modifiable predictor of AKI and non-AKI mortality in emergency hospital admissions | Not AKI population |
| 15 | Salvador, 2023 | A low BUN/creatinine ratio predicts histologically confirmed acute interstitial nephritis | Not AKI population |
| 16 | Senghor, 2022 | Hypoalbuminaemia and Blood Urea Nitrogen/ Creatinine Ratio as Early Markers of Acute Kidney Injury in Postoperative Cardiac Patients- A Prospective Study | Not AKI population |
| 17 | Sun, 2022 | Blood urea nitrogen to creatinine ratio is associated with in‑hospital mortality among critically ill patients with cardiogenic shock | Not AKI population |

**Footnote:** Study with blue underlying is identified from reference screening

**Supplementary Table 3. Subgroup analysis**

| **Variables** | **Subgroups**  **(Number of studies)** | **Sensitivity**  **(95% CI)** | **Specificity**  **(95% CI)** | **P value for subgroup difference** |
| --- | --- | --- | --- | --- |
| BCR cutoff value | Cutoff value 20 | 0.79 (0.51-1.00) | 0.43 (0.03-0.83) | 0.39 for sensitivity  0.42 for specificity |
|  | Other cutoff value | 0.64 (0.16-1.00) | 0.68 (0.20-1.00) |  |
| Study Design | Prospective | 0.80 (0.54-1.00) | 0.44 (0.02-0.86) | 0.26 for sensitivity  0.46 for specificity |
|  | Retrospective | 0.59 (0.07-1.00) | 0.69 (0.12-1.00) |  |
| Chronic kidney disease | Exclusion | 0.83 (0.56-1.00) | 0.35 (0.07-0.77) | 0.25 for sensitivity  0.25 for specificity |
|  | Inclusion | 0.64 (0.25-1.00) | 0.66 (0.29-1.00) |  |

**Supplementary Table 4. QUADAS assessment**

| **Study** | **Test** | **Risk of bias** | | | |  | **Applicability concerns** | | |  |
| --- | --- | --- | --- | --- | --- | --- | --- | --- | --- | --- |
|  |  | **P** | **I** | **R** | **FT** |  | **P** | **I** | **R** | |
| Carvounis, 2002 | BCR | ? | ✓ | ? | ? |  | ✓ | ✓ | ✓ | |
| Dewitte, 2012 | BCR | ? | ✓ | ✓ | ✓ |  | ✓ | ✓ | ✓ | |
| Fushimi, 1990 | BCR | ? | ✓ | ✓ | ✓ |  | ✓ | ✓ | ✓ | |
| Manoeuvrier, 2017 | BCR | ✓ | ✓ | ✓ | ✓ |  | ✓ | ✓ | ✓ | |
| Rachoin, 2012 | BCR | ✓ | ✓ | ✓ | ✓ |  | ? | ✓ | ✓ | |
| Sakr, 2023 | BCR | ✗ | ✗ | ✓ | ✓ |  | ✓ | ✓ | ✓ | |
| Singer, 2012 | BCR | ✓ | ✓ | ✓ | ✓ |  | ✓ | ✓ | ✓ | |
| Slikke, 2020 | BCR | ✓ | ✓ | ✓ | ✓ |  | ✓ | ✓ | ✓ | |
| Uchino, 2012 | BCR | ✓ | ✓ | ✓ | ✓ |  | ✓ | ✓ | ✓ | |

**Footnote:**

P = patient selection; I = index test; R = reference standard; FT = flow and timing.
✓ indicates low risk; ✗ indicates high risk; ? indicates unclear risk.

1. Carvounis, 2002: Patients selected process are unclear about consecutive or random sample. Reference standard are complicated criteria and are unclear whether all patients with pre-renal azotemia are response to therapy. The definition of response to therapy are also unclear therefore, the flow and timing domain is ranked as unclear risk.

2. Fushimi, 1990 & Dewitte, 2012: Patients selected process are unclear about consecutive or random sample. Therefore, we judge it as unclear risk

3. Rachoin, 2012: Unclear acute kidney injury definition, therefore, the domain regarding the applicability concerns are unclear risk

4. Sakr, 2023: Case-control design and divided patients according to acute kidney injury (yes or no) and BUN/Cr level (high or low)

5. Sakr, 2023: Using best cutoff value but not pre-specific cutoff value

**Supplementary Table 5. Summary of Certainty of Evidence Assessment for pre-renal azotemia/transient AKI**

| **Sensitivity** | **0.74 (95% CI: 0.43 to 0.91)** |
| --- | --- |
| **Specificity** | **0.52 (95% CI: 0.21 to 0.82)** |

| **Prevalences** | **40%** | **47%** | **54%** |
| --- | --- | --- | --- |

| **Outcome** | **№ of studies (№ of patients)** | **Study design** | **Factors that may decrease certainty of evidence** | | | | | **Effect per 1,000 patients tested** | | | **Test accuracy CoE** |
| --- | --- | --- | --- | --- | --- | --- | --- | --- | --- | --- | --- |
|  |  |  | **Risk of bias** | **Indirectness** | **Inconsistency** | **Imprecision** | **Publication bias** | **pre-test probability of 40%** | **pre-test probability of 47%** | **pre-test probability of 54%** |  |
| **True positives** (patients with pre-renal AKI/Transient AKI) | 6 studies 731 patients | cross-sectional (cohort type accuracy study) | not serious | not serious | very serious^a^ | not serious | none | 296 (172 to 364) | 348 (202 to 428) | 400 (232 to 491) | ⨁⨁◯◯ Low^a^ |
| **False negatives** (patients incorrectly classified as not having pre-renal AKI/Transient AKI) |  |  |  |  |  |  |  | 104 (36 to 228) | 122 (42 to 268) | 140 (49 to 308) |  |
| **True negatives** (patients without pre-renal AKI/Transient AKI) | 7 studies 772 patients | cross-sectional (cohort type accuracy study) | not serious | not serious | very serious^a^ | not serious | none | 312 (126 to 492) | 276 (111 to 435) | 239 (97 to 377) | ⨁⨁◯◯ Low^a^ |
| **False positives** (patients incorrectly classified as having pre-renal AKI/Transient AKI) |  |  |  |  |  |  |  | 288 (108 to 474) | 254 (95 to 419) | 221 (83 to 363) |  |

**Footnote**

^a^. High heterogeneity

**Supplementary Table 6. Summary of Certainty of Evidence Assessment for non-dialysis requiring AKI**

| **Sensitivity** | **0.49 (95% CI: 0.44 to 0.55)** |
| --- | --- |
| **Specificity** | **0.71 (95% CI: 0.64 to 0.77)** |

| **Prevalences** | **97%** | **90%** | **80%** |
| --- | --- | --- | --- |

| **Outcome** | **№ of studies (№ of patients)** | **Study design** | **Factors that may decrease certainty of evidence** | | | | | **Effect per 1,000 patients tested** | | | **Test accuracy CoE** |
| --- | --- | --- | --- | --- | --- | --- | --- | --- | --- | --- | --- |
|  |  |  | **Risk of bias** | **Indirectness** | **Inconsistency** | **Imprecision** | **Publication bias** | **pre-test probability of 97%** | **pre-test probability of 90%** | **pre-test probability of 80%** |  |
| **True positives** (patients with non-dialysis requiring AKI) | 5 studies 14494 patients | cohort & case-control type studies | serious^a^ | not serious | very serious^b^ | not serious | none | 475 (427 to 534) | 441 (396 to 495) | 392 (352 to 440) | ⨁◯◯◯ Very low^a,b^ |
| **False negatives** (patients incorrectly classified as not having non-dialysis requiring AKI) |  |  |  |  |  |  |  | 495 (436 to 543) | 459 (405 to 504) | 408 (360 to 448) |  |
| **True negatives** (patients without non-dialysis requiring AKI) | 5 studies 429 patients | cohort & case-control type studies | serious^a^ | not serious | very serious^b^ | not serious | none | 21 (19 to 23) | 71 (64 to 77) | 142 (128 to 154) | ⨁◯◯◯ Very low^a,b^ |
| **False positives** (patients incorrectly classified as having non-dialysis requiring AKI) |  |  |  |  |  |  |  | 9 (7 to 11) | 29 (23 to 36) | 58 (46 to 72) |  |

**Footnote**

^a.^ Sakr, 2023: risk of bias regarding patient selection (case-controlled study) and used best cutoff value

^b.^ high heterogeneity

**Supplementary Figure 1. PRISMA flow chart**

**Supplementary Figure 2. Forest plot of BCR diagnostic accuracy for transient AKI: sensitivity and specificity**

**Supplementary Figure 3. Forest plot of BCR diagnostic accuracy for transient AKI: positive likelihood ratio and negative likelihood ratio**

**Supplementary Figure 4. SROC curves of Blood UCR for prediction of Transient AKI**

**Supplementary Figure 5. Likelihood ratio scattergram of BCR for transient AKI**

**Supplementary Figure 6. Likelihood ratio scattergram of BCR for non-dialysis requiring AKI**

**Supplementary Figure 7.** Sensitivity and specificity of UCR for non-dialysis requiring AKI: Sensitivity Analysis

**Supplementary Figure 8.** Positive likelihood ratio and negative likelihood ratio of UCR for non-dialysis requiring AKI: Sensitivity Analysis

**Supplementary Figure 9. Forest plot of UCR vs FeNA for pre-renal azotemia/transient AKI**

**Supplementary Figure 10. SROC curves of Blood UCR vs FeNA for prediction of Pre-renal Azotemia/Transient AKI**

**Supplementary Figure 11. Funnel plot for pre-renal azotemia/transient AKI**

**Supplementary Figure 12. Funnel plot for non-dialysis requiring AKI**

**Supplementary Figure 13. Summary of risk of bias assessment**

**Supplementary Figure 14. Fagan's Nomogram for Blood UCR as Pre-renal Azotemia/Transient AKI Prediction Marker with Pre-test probability 45% (A) and 55% (B)**

**Supplementary Figure 15. Fagan's Nomogram for Blood UCR as Non-dialysis Requiring AKI Prediction Marker with Pre-test probability 85% (A) and 95% (B)**
